# Molecular Properties of Globin Channels and Pores: Role of Cholesterol in Ligand Binding and Movement

**DOI:** 10.3389/fphys.2016.00360

**Published:** 2016-09-05

**Authors:** Gene A. Morrill, Adele B. Kostellow

**Affiliations:** Department of Physiology and Biophysics, Albert Einstein College of MedicineBronx, NY, USA

**Keywords:** Globins, band-3 protein, cholesterol, protein cavities, channels, ligands

## Abstract

Globins contain one or more cavities that control or affect such functions as ligand movement and ligand binding. Here we report that the extended globin family [cytoglobin (Cygb); neuroglobin (Ngb); myoglobin (Mb); hemoglobin (Hb) subunits Hba(α); and Hbb(β)] contain either a transmembrane (TM) helix or pore-lining region as well as internal cavities. Protein motif/domain analyses indicate that Ngb and Hbb each contain 5 cholesterol- binding (CRAC/CARC) domains and 1 caveolin binding motif, whereas the Cygb dimer has 6 cholesterol-binding domains but lacks caveolin-binding motifs. Mb and Hba each exhibit 2 cholesterol-binding domains and also lack caveolin-binding motifs. The Hb αβ-tetramer contains 14 cholesterol-binding domains. Computer algorithms indicate that Cygb and Ngb cavities display multiple partitions and C-terminal pore-lining regions, whereas Mb has three major cavities plus a C-terminal pore-lining region. The Hb tetramer exhibits a large internal cavity but the subunits differ in that they contain a C-terminal TM helix (Hba) and pore-lining region (Hbb). The cavities include 43 of 190 Cygb residues, 38 of 151 of Ngb residues, 55 of 154 Mb residues, and 137 of 688 residues in the Hb tetramer. Each cavity complex includes 6 to 8 residues of the TM helix or pore-lining region and CRAC/CARC domains exist within all cavities. Erythrocyte Hb αβ-tetramers are largely cytosolic but also bind to a membrane anion exchange protein, “band 3,” which contains a large internal cavity and 12 TM helices (5 being pore-lining regions). The Hba TM helix may be the erythrocyte membrane “band 3” attachment site. “Band 3” contributes 4 caveolin binding motifs and 10 CRAC/CARC domains. Cholesterol binding may create lipid-disordered phases that alter globin cavities and facilitate ligand movement, permitting ion channel formation and conformational changes that orchestrate anion and ligand (O_2_, CO_2_, NO) movement within the large internal cavities and channels of the globins.

## Background

Globins are small respiratory proteins that reversibly bind O_2_ by means of an iron-containing porphyrinring. Globin-like proteins have been identified in bacteria, plants, fungi, and animals (Hardison, 1996, 1998) and contain internal cavities and packing anomalies that appear to reduce thermodynamic stability but may actually provide interior pathways for the diffusion of ligands (Brunori and Gibson, 2001; Teeter, 2004; Brunori et al., 2005; Olson et al., 2007). The pathways and mechanism of movement of ligands within protein cavities and tunnels have been studied by spectroscopy, by crystallography, and by stimulation as well as by mutagenesis mapping experiments (e.g., Tomita et al., 2009; Salter et al., 2012) and have been characterized using molecular dynamics simulations (e.g., Paramo et al., 2014). Studies of myoglobin suggest that thermally or photo-dissociated ligands first migrate into open spaces within the globin interior and then diffuse back to the distal pocket, where they either rebind to the iron or escape from the protein through a gate regulated by motions of His(E7)64 (reviewed in Tomita et al., 2009). Salter et al. (2012), applying a mutagenesis mapping strategy to the neuronal mini-hemoglobin from *Cerebratulus lacteus*, identified multiple internal pathways for O2 entry into and exit from the globin.

Studies on structural and functional variation within the globin superfamily adds evidence to the hypothesis that hemoglobin and myoglobin are two specialized heme proteins within a broad evolutionary family that evolved enabling larger and more efficient animals (Richter et al., 2009). Cytoglobin (Cygb) is a more recently identified intracellular globin that i s expressed ubiquitously in low levels in human tissues and exists as a dimer (reviewed in Pesce et al., 2002). Neuroglobin (Ngb) is monomeric and is expressed in specific areas of the brain, notably the frontal lobe, subthalamic nucleus, and thalamus (Mammen et al., 2002; Pesce et al., 2002), although at μM concentrations. Myoglobin (Mb) is a monomeric protein found in muscle tissue of vertebrates in general and it is related to the α-subunit of hemoglobin (Hb; Teeter, 2004). Hb is a tetramer (ABAB) containing both α and β subunits. In mammals it makes up about 96% of the red blood cells' dry content (by weight; Weed et al., 1963). Neurons also express hemoglobin α (Hba) and β (Hbb) chains in both rat and human brains (Richter et al., 2009). Hba and Hba-like molecules are also found in many invertebrates, fungi, and plants (e.g., Weber and Vinogradov, 2001). *In silico* analysis of the genome of *C. elegans* identified 33 putative globin genes and, as noted by the authors, it is a “mystery why such a tiny animal needs so many globins” (Hoogewijs et al., 2007). Identification of additional globin types (GbX, GbE, and GbY) with unknown physiological functions (cit. Burmeister and Hankeln, 2014) has further complicated understanding of the globin gene family evolution. Phylogenetic analysis of vertebrate globins indicate that erythroid-specific globin has independently evolved O_2_−transport functions in different lineages (Burmeister and Hankeln, 2014). It has also become evident that vertebrate, plant, and other metazoan Hb's with a classical 3/3 α-helical fold may share a common ancestor with one of three bacterial globin types (Laberge and Yonetani, 2007; Burmeister and Hankeln, 2014).

When erythrocytes are hydrolyzed and the hemolysate rinsed, some Hb remains attached to a bicarbonate/chloride exchanger “band 3” membrane protein, a “multipass” integral component of most cell membranes (e.g., Chu et al., 2008). The nature of the attachment of Hb to “band 3” is not defined. “Band 3” is an important structural protein of the erythrocyte cell membrane, making up 25% of the cell membrane surface (Chu et al., 2008) but has not been associated with globins other than hemoglobin. However, its wide cellular distribution suggests that “band 3” may play an important anion exchanger role in heme systems other than the erythrocyte.

In this study, we analyze the internal cavities and/or channels using computation Methods such as the PoreWalker algorithm (Pellegrini-Calace et al., 2009) and compare the cavity topology with that of the transmembrane helices, the pore-lining regions (Nugent and Jones, 2012), and the cholesterol binding (CRAC/CARC) domains (Li and Papadopoulos, 1998; Baier et al., 2011; Fantini et al., 2016) in closely related members of the extended *Homo sapiens* globin family: Cygb, Ngb, Mb, and Hb subunits α and β. We find that each globin contains either one transmembrane helix or one pore-lining channel-forming amino acid sequence in the C-terminal region as well as one or more internal cavities. The pore-lining regions, TM helices, and cavities coexist in combination with caveolin-binding motifs and/or cholesterol-binding (CRAC/CARC) motifs that provide an environment in which membrane cholesterol levels regulate ion channel formation.

## Materials and methods

### Materials

The amino acid sequences of *H. sapiens* cytoglobin (Accession #Q8WWM9), neuroglobin (Accession #Q9NPG2), myoglobin (Accession #P02144), and hemoglobin subunits alpha (Accession #P69905), and beta (Accession #P68871) were downloaded from the ExPASy Proteonomic Server of the Swiss Institute of Bioinfomatics (http://www.expasy.org; http://www.uniprot.org). About 98% of the protein sequences provided by UniProtKB are derived from the translation of the coding sequences (CDS) which have been submitted to the public nucleic acid databases, the EMBL-Bank/Genbank/DDBJ databases (INSDC). Amino acid sequences were compared using the Pairwise Sequence Alignment software (LALIGN) at http://www.ebi.ac.uk/Tools/services/weblalign/ to find internal duplications by calculating non-intersecting local alignments. The Emboss Water protocol (version 36.3.5e Nov, 2012; preload8) used here employs the Smith-Waterman algorithm (with modified enhancements) to calculate the local alignment of two sequences.

### Secondary structure, transmembrane helix, and pore-lining region predictions

PSIPRED is a simple and accurate secondary structure prediction method, incorporating two feed-forward neural networks which perform an output obtained from Position Specific Iterated—Blast. PSIPRED 3.2 achieves an average Q3 score of 81.6% (Nugent et al., 2011) and is available at http://bioinf.cs.ucl.ac.uk/psipred/. Alpha-helical transmembrane (TM) channel proteins play key roles in a variety of cellular processes essential in movement of ions and molecules across membrane lipid bilayers. As noted by Nugent and Jones (2012), technical difficulties in obtaining high quality crystals have led to an under-representation of channel proteins in structural databases. These workers have trained a support vector machine classifier to predict the likelihood of a TM helix being involved in pore formation. This approach has a prediction accuracy of 72%, while a support vector regression model is able to predict the number of subunits participating in the pore with 62% accuracy. Pore-lining regions in transmembrane protein sequences in this study were predicted using the method of Nugent and Jones (2012).

The Consensus Constrained TOPology prediction (CCTOP; http://cctop.enzim.ttk.mta.hu) server is a web-based application providing transmembrane topology prediction. In addition to utilizing 10 different state-of-the-art topology prediction methods, the CCTOP server incorporates topology information from existing experimental and computational sources available in the PDBTM, TOPDB, and TODOM data bases using the probabilistic framework of hidden Markov model (Dobson et al., 2015).

Based on X-ray crystallography data (PDB files), Pellegrini-Calace et al. (2009) have developed a computational approach (PoreWalker 1.0) for the identification and characterization of channels and cavities in proteins based on their three-dimensional structure. The software is available as a web-based resource at http://www.ebi.ac.uk/thornton-srv/software/PoreWalker/.

### TMKink: A method to predict transmembrane helix kinks

Meruelo et al. (2011) have identified distinct residue preferences in kinked vs. non-kinked helices and have exploited these differences and residue conservation to predict kinked helices using a neural network algorithm. The kink predictor, TMKink, is available at http://tmkinkpredictor.mbi.ucla.edu/.

### Caveolin-binding motifs and scaffolding domains

Using a GST-fusion protein containing the caveolin scaffolding domain as a receptor to select peptide ligands from a bacteriophage display library, Covet et al. (1997) identified at least three related but distinct caveolin binding motifs, ΦxxxxΦxxΦ, ΦxΦxxxxΦ, and ΦxΦxxxxΦxxΦ (where Φ represents an aromatic amino acid, W, Y, or F), that have been shown to interact with caveolin in most proteins. The caveolin scaffolding domain is ascribed a role in the recognition and binding of cholesterol, which is highly concentrated in caveolae and essential for their formation. More precisely, formation of caveolae strictly requires tight binding of caveolin to cholesterol (reviewed in Covet et al., 1997). This functionality is thought to localize to a cholesterol recognition/interaction amino acid consensus motif in residues VTKYWFYR (reviewed in Fantini and Barrantes, 2013) defined as the CRAC motif. Despite its short length, this 20-residue segment appears to incorporate an array of critical functionalities in overlapping sequence elements.

### The CRAC/CARC domains

CRAC is a short linear amino acid motif that mediates binding to cholesterol and stands for Choles-terol Recognition/Interaction A mino acid Consensus sequence (Li and Papadopoulos, 1998; reviewed in Fantini and Barrantes, 2013). In a C-terminus to N-terminus direction the motif consists of a polar Leu (L) or Val (V) residue, followed by a segment containing 1–5 of any residues, followed by a mandatory aromatic Tyr (Y) residue, a segment containing 1–5 of any residues, and finally a basic Lys (K) or Arg (R). In the one letter amino acid codes the algorithm is (L/V) – X1–5 – (Y) – X1–5 – (K/R). A second cholesterol recognition domain similar to the CRAC domain (CARC) has been identified (Baier et al., 2011) but exhibits the opposite orientation along the polypeptide chain (“in- verted CRAC”), i.e., (K/R) – X1–5 – (Y/F) – X1–5 – (L/V). CARC is distinct from CRAC in that the central amino acid can be either Y or F.

## Results

### Pore-lining regions and transmembrane helices in Cytg, Ngb, Mb, and Hb subunits α and β

Transmembrane (TM) helices and pore-lining regions span a large variety of membrane systems including cell-surface receptors, redox proteins, ion channels, and transporters (reviewed in Hildebrand et al., 2006). TM helices are found to be amino acid sequences with hydrogen-bonded helical configurations, including α-, 3_10_− and π-helices. The α-helix is very common; while the 3_10_ helix is found at the ends of the α-helix and π-helices are rare. Based on data for 160 transmembrane helices of 15 non-homologous high resolution X-ray protein structures, Hildebrand et al. (2004) found the average TM helix to be a 17.3 ± 3.1 (*SD N* = 160) residues in length. However, it is difficult to obtain a representative sampling of the membrane proteins.

Many membrane proteins have proven to be difficult to crystallize owing to their partially hydrophobic surfaces, flexibility, and lack of stability as well as physiologically bound lipids (reviewed in Carpenter et al., 2008).

**Figure 1:**
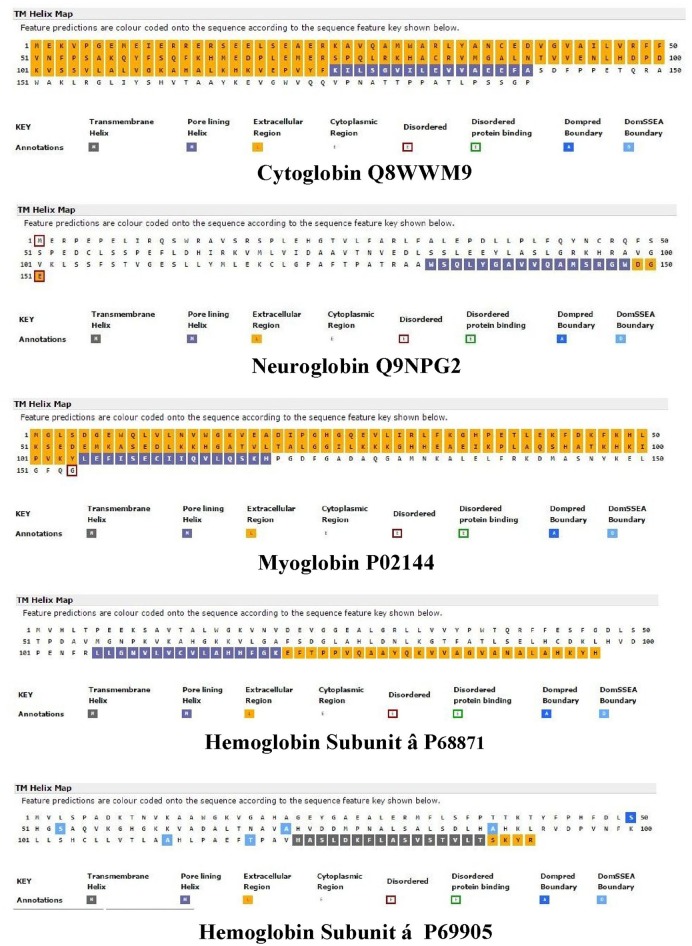
**An analysis of the topology of ***Homo sapiens*** cytoglobin (upper plot, Accession #Q8WWM9), neuroglobin (second down, Accession #Q9NPG2), myoglobin (third down, Accession #P02144), hemoglobin subunit β (forth down, Accession #P68871), and hemoglobin subunit α (lower plot, Accession #P69905) using the support vector machine-based TM topology predictor MEMSAT-SVM (see Section Methods)**. Transmembrane pore-lining regions are highlighted in blue, whereas transmembrane helices are highlighted in black. White sequences indicate predicted cytoplasmic regions; those highlighted in orange represent extracellular regions. Amino acid sequences are those published in the Swiss Protein Knowledgebase (www.uniprot.org). See Section Methods for details.

Nugent et al. (2011) have developed a method (MEMSAT-SVM) to predict pore-lining regions in transmembrane (TM) proteins and to distinguish pore-lining regions from TM helices (Nugent and Jones, 2012). Pore-lining regions are usually enriched in negatively (e.g., E, D) or positively charged residues (e.g., K, R, H). Four features were used to train a support vector regression model: sequence length, the number of pore lining residues, with the target value set to the number of subunits contacting the pore within the membrane region. Nugent et al. (2011) were able to identify pore-lining regions and to predict both the likelihood of transmembrane helices involved in pore (channel) formation and to determine the number of subunits required to form a complete pore or channel. As shown in Figure 1, topological analyses of *H. sapiens* Cygb (top), Ngb (upper middle), Mb (middle), Hbb (subunit β, lower middle), and Hba (subunit α, bottom) predict that, with the exception of Hba, each globin contains a 16 residue pore-lining (channel-forming) structure in the C-terminal region of the protein (blue squares). In contrast, Hba contains a 15 residue TM helix (black squares). Typically, channel proteins contain a cavity (or pore) which spans the entire membrane protein with an opening on each side of the membrane. The pore often runs parallel to TM helices, forming a path along which ions or molecules travel, with adjacent structural features determining pore specificity. As shown in Figure 1, the N-terminal region of both Cygb and Mb was predicted to be extracellular (orange boxes) whereas the N-terminal region of Ngb and the Hba and Hbb subunits is cytoplasmic (white boxes).

**Figure 2:**
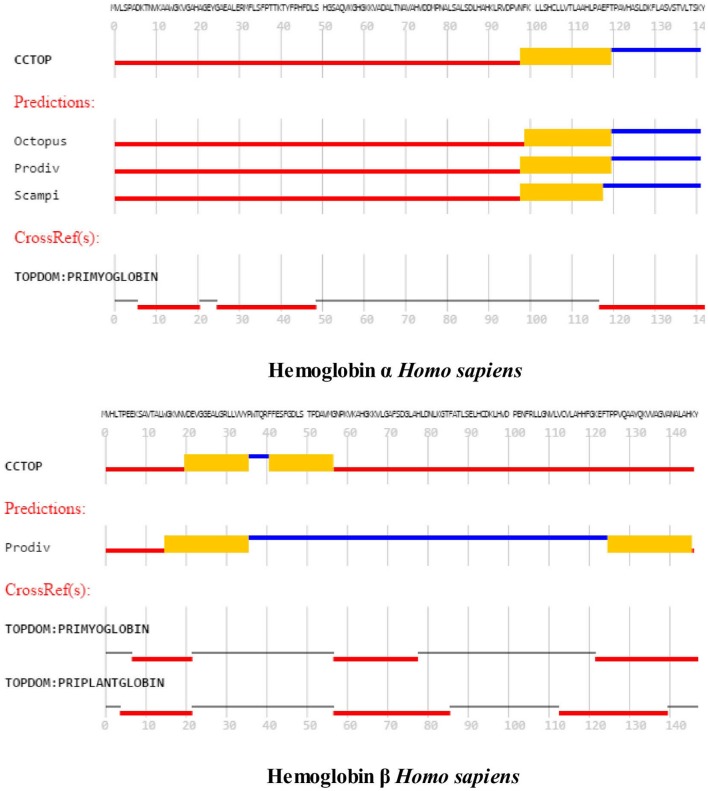
**An analysis of the topology of ***Homo sapiens*** hemoglobin subunit α (upper panel) and hemoglobin subunit β (lower panel) using the Consensus Constrained TOPology prediction web server (see Section Methods)**. The Figure 1 shows the amino acid sequence as colored by the consensus topology. Colors are based on the localization: gray (transit sequence), black (signal peptide), blue (extracytosolic), red (cytosolic), yellow (membrane), and orange (re-entrant loop regions).

Figure 2 compares the topology for Hba (upper panel) and Hbb (lower panel) using the Consensus Constrained TOPology server (Dobson et al., 2015). Colors are based on the localization of peptide sequences: gray (transit sequence), black (signal peptide), blue (extracytosolic), red (cytosolic), yellow (transmembrane), and orange (re-entrant loop regions). CCTOP analysis of Hba predicts a TM helix in the 100–120 residues region, consistent with that shown by the MEMSAT-SVM algorithm in Figure 1. Similar TM helices were predicted by the Octopus, Prodiv, and Scampi algorithms. In contrast, CCTOP analysis of Hbb predicted a helix-turn-helix in the 20–56 residue region but failed to detect pore-lining regions in the C-terminal region. The findings in Figures 1, 2 suggest that Cygb, Ngb, Hba, and Hbb, as well as Hba (Shaklai et al., 1977), may also be integral to cell membranes.

Cybg, Ngb, Mb, Hba, and Mbb amino acid sequences were compared using LALIGHN (see Section Methods). As shown in Table 1, a comparison of *H. sapiens* Cybg and Ngb indicates a 29.4% identity (63.4% similar) in a 154 amino acid overlap (19–171: 3–152) with a Waterman:Eggert score of 251. A similar comparison of Cybg and Mb indicates a 28.9% identity (63.1% similar) in a 149 amino acid overlap (19–167: 3–142) with a Waterman:Eggert score of 227. A comparison of Mb and Hba indicates a 39.3% identity (46.4% similar) with an amino acid overlap of only 28 amino acids (17–39: 57–84) with a Waterman-Eggert score of only 41. Finally, a comparison of Hba and Hbb indicated a 25.5% identity (58.6% similar) in a 145 amino acid overlap (4–146:3–147) with a Waterman-Eggert score of 161. Despite the limited amino acid sequence identities and similarities shown here, all four clearly belong to the globin superfamily (Pesce et al., 2002), all contain the typical globin fold (Laberge and Yonetani, 2007) and reversibly bind O2, CO, and NO (reviewed in Pesce et al., 2002). Hb and Mb have been considered almost exclusively as transporters of metabolic gases and nutrients whereas erythrocytes also take up and inactivate endothelium-derived NO to form methemoglobin and nitrate. As noted by Brunori et al. (2005), Ngb is unlikely to be involved in O2 transport (like Mb) but has been proposed to act as a sensor of the O2/NO ratio in the cell, possibly regulating the GDP/GTP exchange rate, forming a specific complex with the Gα*βγ*–protein when oxidized, but not when bound to a gaseous ligand (reviewed in Chu et al., 2008). Tomita et al. (2009), using pulsed- laser at cryogenic temperatures to study CO migration in Mb found that the migration of the CO molecule into each cavity induces structural changes of the amino acid residues, resulting in the sequential motion of the ligand, and the cavity. They propose that a breathing motion of internal cavities is a general mechanism of ligand migration.

**Table 1:** **LALIGN Analysis of ***Homo sapiens*** Extended Globin Family: Comparison of Cytoglobin, Neuroglobin, Myoglobin, and Hemoglobin Subunits α and β**.

**LALIGN analysis**	**Cyg vs. Ngb**	**Cyg vs. Mb**	**Mb vs. Hba**	**Hba vs. Hbb**
% Identical	29.4	28.9	39.3	25.5
% Similar	63.4	63.1	46.4	58.6
Amino acid overlap	154 aa 19–171:3–152	149 aa 19–167:3–142	28 aa 17–39:57–84	145 aa 4–146:3–147
Waterman–Eggert score	251	227	41	161

### Identification and characterization of channels and cavities in Cytg, Ngb, Mb, and Hb

Pellegrini-Calace et al. (2009) have developed an improved computational approach (PoreWalker 1.0) for the identification and characterization of channels and cavities in proteins, based on their three-dimensional structure. Given a set of 3D crystallography coordinates, this method can detect and identify the pore centers and axis using geometric criteria and the biggest and longest cavity/channel through the protein is identified. Pore features, including diameter profiles, pore-lining residues, the size, shape, and regularity of the pore are used to provide a quantitative and visual characterization of the channel. Since pore-forming proteins interact with lipid bilayers to generate “proteolipidic” pores (Gilbert et al., 2014), the “crystal structure” of a lipid-free protein crystal may not correspond precisely to the physiological cavity/channel.

**Figure 3:**
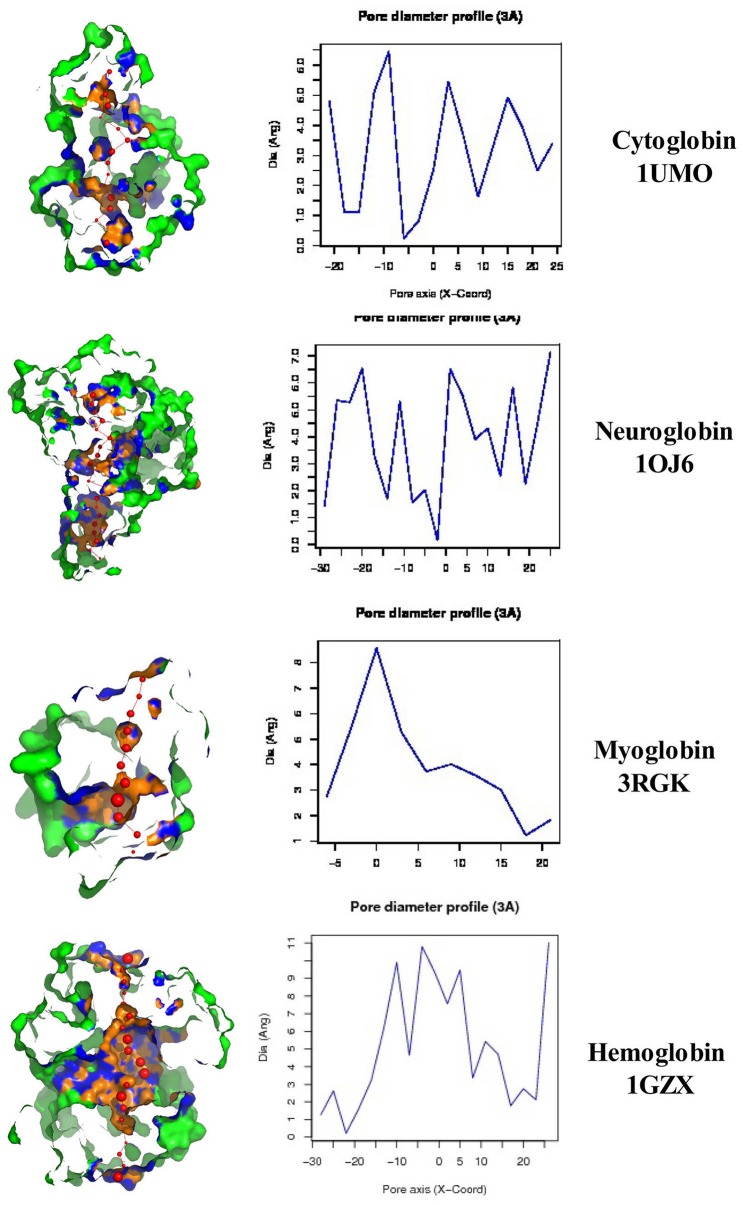
**PoreWalker output for ***Homo sapiens*** cytoglobin (1UMO, top), neuroglobin (IOJ6, second down), myoglobin (3RGK, third down), and hemoglobin (1GZX, bottom)**. Left column is a visualization of a pore section showing pore-lining residues and pore centers at 3À steps; the section of the structure was obtained by cutting the protein structure along the *xy*-plane, where the *x*-axis corresponds to the pore axis, and *y*-coordinate >0 only are displayed. The pore-lining atoms and residues are colored in orange and blue, respectively. The remaining portion of the protein is shown in green. The plots in the right column illustrate the pore diameter profiles at 3À steps.

The left column of Figure 3 illustrates the PoreWalker structure profiles for Cygb (top), Ngb (upper middle), Mb (lower middle), and Oxy T State Hb (bottom), whereas the right column presents the corresponding pore diameter profiles. The structure profiles are visualizations of a pore section showing pore-lining residues and pore centers, using 3À steps: the section of each structure was obtained by cutting the protein structure along the *xy*-plane, where the *x*-axis corresponds to the pore axis, and the *y*-coordinate >0 only are displayed. The PoreWalker graphics identifies and characterizes the biggest and longest cavity in a set of 3D crystallography coordinates in a cholesterol and lipid-free preparation. The pore-lining atoms and residues are colored in orange and blue, respectively. The remainder of the protein is shown in green. Red spheres represent pore centers at given pore heights and their diameters correspond to 1/10 of the pore diameter calculated at that point. As can be seen in the right hand column, a cavity of varying diameters extends across all four globins. Cygb (1UMO) and Ngb (1OJ6) each contain a sequence of cavities with varying diameters, whereas Mb (3RGK) exhibits a large single cavity localized left (Mb) whereas Oxy Hb (1GZX) contains a large centrally located cavity(s).

Maximum cavity diameters varied from about 6 to 11 angstroms in the 4 globin crystals, or about 3–5 times the diameter of a water molecule. The cavities and channels may be larger in fully hydrated proteins embedded in lipid bilayers.

### Putative domain boundaries in human Cygb, Ngb, Mg, and Hbb

The shortest sequence of amino acids in proteins that contains functional and structural information is termed a “motif,” whereas a conserved part of a given protein that can evolve, function, and exist independently is termed a “domain.” Domains and motifs contribute to the structural basis of the physiological functions of proteins and each domain can be considered as a semi-independent structural unit of a protein capable of folding independently (e.g., Batey et al., 2008; Dill et al., 2008; Pang et al., 2008). A variety of different methodologies have been employed to predict domains but many are fraught with problems since domain assignment is difficult even when the structures are known. Bryson et al. (2007) have developed a useful method for computer-assisted protein domain boundary prediction, using the DomPred server (see Section Methods). This server uses the results from two completely different categories (DPS and DomSSEA). Each is individually compared against one of the latest domain prediction benchmarks to determine their respective reliabilities.

**Figure 4:**
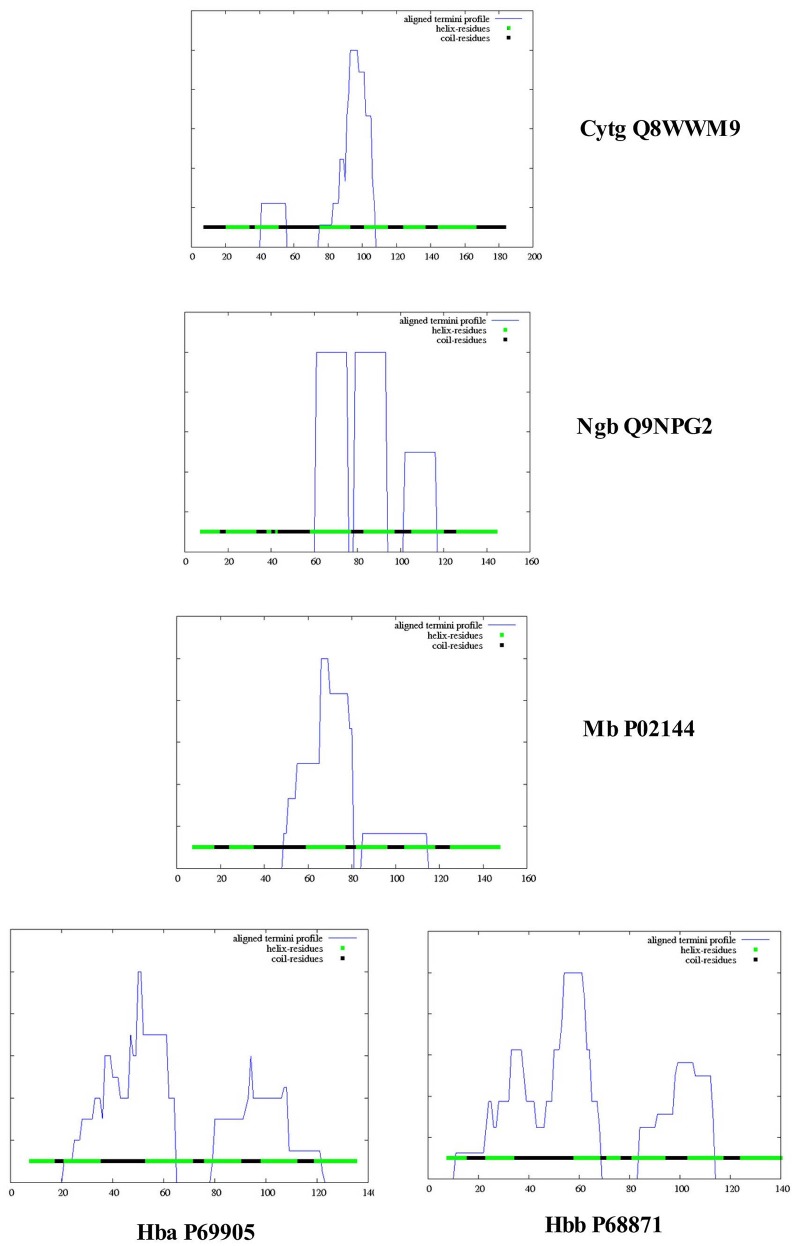
**Protein domain and motif sequence analysis of ***Homo sapiens*** cytoglobin (Cybg Q8WWM9, top), neuroglobin (Ngb Q9NPG2, second from top), Myoglobin (Mb P02144, third from top), hemoglobin subunit α (Hba P69905, bottom left), and hemoglobin subunit β (Hbb P68871, bottom right) as predicted by the DomPred algorihm**.

**Figure 5:**
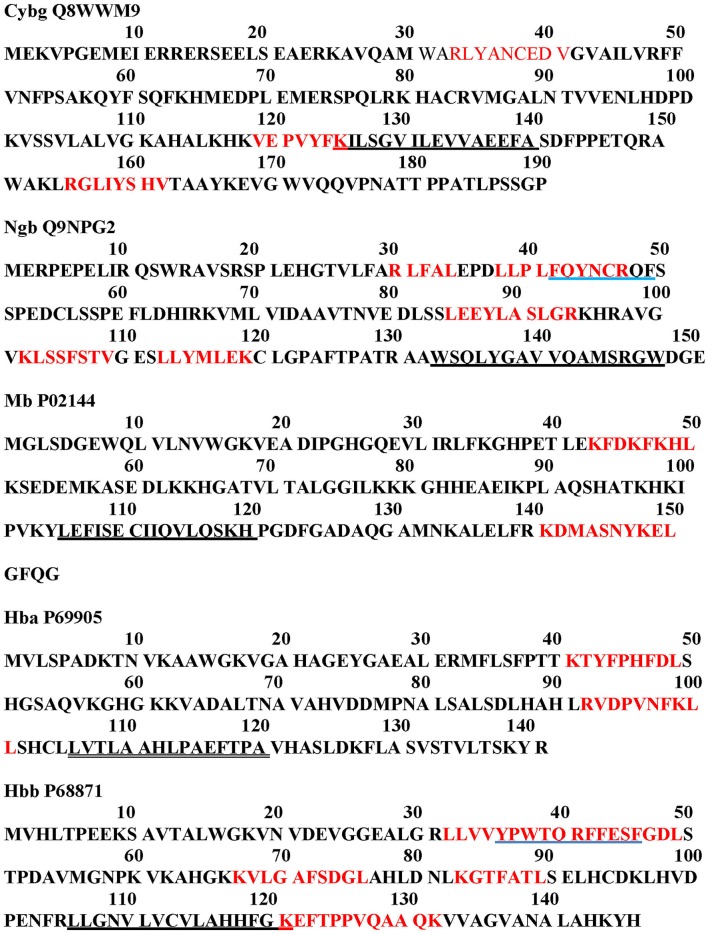
**Cholesterol-binding (CRAC/CARC) motif sequence analysis (red highlights) and caveolin distribution (blue underline) in ***Homo sapiens*** cytoglobin (Cybg Q8WWM9, top), neuroglobin (Ngb Q9NPG2, second from top), Myoglobin (Mb P02144, third from top), hemoglobin subunit α (Hba P69905, second from bottom), and hemoglobin subunit β (Hbb P68871, bottom)**. CRAC/CARC motifs are indicated by red highlighting and caveolin binding motifs are underlined in blue. Pore-lining regions are single underlined in black. TM helices are indicated by double underlining.

Figure 4 illustrates a DomPred analysis of the five different globins from *H. sapiens*. As can be seen, the protein domain patterns differ, with the domains of Hba and Hbb being the most complex. As noted by Marsden et al. (2002), the modular nature of many domains means that they can often be found in proteins with a similar content, but with different orders, or in different proteins in combination with entirely different domain structures. In addition to the TM helices, pore-lining regions, and domain structures described above, functional domains such as CRAC or CARC cholesterol binding motifs (reviewed in Fantini and Barrantes, 2013) can be identified within the Cygb, Ngb, Mb, and Hb subunits α (Hba) and β (Hbb). Figure 5 compares the distribution of cholesterol-binding (CRAC/CARC) domains (highlighted in red) with caveolin-binding motifs (underlined in blue) and both TM helices (double underlined) and pore-lining regions (single underlined) identified. A cholesterol binding domain similar to CRAC has also been characterized and is termed a “CARC” motif (reviewed in Fantini and Barrantes, 2013). The so- called “CARC motif” is comparable to the CRAC domain but exhibits the opposite orientation along the polypeptide chain (“inverted CRAC”; Baier et al., 2011) and is distinct from CRAC in that the central amino acid can be either Y or F.

Molecular modeling studies have shown that CRAC/CARC motifs have a good fit for cholesterol (cit. Fantini and Barrantes, 2013). Although cholesterol is concentrated in sphingolipid-enriched membrane microdomains such as “lipid rafts” (Silvius, 2003), it is also present in the lipid disordered phase of the plasma membrane that contains high amounts of glycerolphospholipids such as phosphatidylcholine (Heberle and Feigenson, 2011). Artificial lipid membranes also contain rather large voids, the volume of which can be an order of magnitude larger than the largest spherical cavities present in biological membranes (Alinchenko et al., 2005). Studies have shown that addition of cholesterol to artificial lipid membranes produces elongated voids lying perfectly parallel with the membrane in normal axis (Alinchenko et al., 2005), reminiscent of the cavities shown for globins (see Figure 3).

### Cholesterol-binding sites within globin cavities and channels

Since the PoreWalker algorithm identifies both the amino acid residues and/or domains lining the largest cavity within each globin, it is possible to identify potential cholesterol binding (CRAC/CARC) sites lining the globin cavities. Caveolin (blue underline in Figure 5) is an unusual protein that can exist both as an integral membrane protein and as a soluble protein in multiple cell compartments (reviewed in Liu et al., 2002). As shown, Ngb and Hbb each contain a caveolin binding motif (^42^FQYNCRQF^49^ and ^36^YPWTQRFFESF^46^) overlapping CRAC/CARC motifs. Topological analysis of all three isoforms of *H. sapiens* caveolin indicates that each caveolin molecule also contains a pore-lining region, a TM helix-turn-TM helix, and a CRAC/CARC motif (Morrill et al., 2014). Thus, the presence of CRAC/CARC motifs as well as pore-lining regions within the caveolins in proximity to either the pore-lining regions or TM helices in Ngb and Hba could contribute to channel formation.

As shown in Figure 5, two to six CRAC/CARC domains are associated with the Cybg, Ngb, Mb, Hba, and Hbb monomers. Only Cybg (top) and Hbb (bottom) exhibit pore-lining regions with minimal (one residue) overlapping CRAC/CARC motifs. The Cybg dimer would contain up to 6 bound cholesterol molecules whereas the Hb tetramer would contain up to 14 bound cholesterols. Judging from the topology for Cybg and Hbb shown in Figure 1, the CRAC/CARC motifs are at the protein surface and may involve surface docking sites (e.g., Listowski et al., 2015). However, since removal of cholesterol and/or other lipids is often essential in obtaining protein crystals (reviewed in Carpenter et al., 2008), the protein data base for globin preparations used in x-ray crystallographic analysis list only globin, heme and sulfate or phosphate ions. Cholesterol, phospholipids and water in kinks have been removed.

Similarly, it should be noted that Hb used in biophysical studies (e.g., Roche et al., 2013) has been prepared by anion exchange chromatography methods (Andrade et al., 2004) that largely removes endogenous lipids and may thus alter the physiological properties of the Hb molecule.

It should also be noted that methionine is the N-terminal amino acid in each of the 5 globins shown in Figure 5. In a biological system, protein synthesis is initiated universally with the amino acid methionine (see Drabkin and RajBhandary, 1998) which is subsequently removed by enzymes in the cell. Since x-ray crystallography methods require large quantities of a pure protein (Watanabe et al., 2010), crystallography pdb files of cell-free preparations such as those analyzed by PoreWalker (Figure 3) contain N-terminal methionine. The codon initiator methionine is therefore listed in Figure 5, although it may not be present physiologically. A contribution from N-terminal methionine to the crystallographic structure has not been ruled out.

### Correlation of glycosylation with cholesterol binding (CRAC/CARC) motifs

Most plasma membrane and secretory proteins contain one or more carbohydrate chains and the addition and subsequent processing of carbohydrates is the principal chemical modification of such proteins (Cit. Moremen et al., 2012). Glucose reacts non-enzymatically with α- and ε-amino groups on proteins *in vitro* and was shown to occur *in vivo* with the discovery of a naturally existing human Hb component, Hb A1c (Shapiro et al., 1980). Hb glycosylation is elevated in patients with diabetes mellitus, reflective of the increased blood glucose levels. The major Hb glycosylation sites *in vitro* and *in vivo* include β-Lys(K)-66 and β-Lys(K)-120. As shown in Figure 5, after correcting for the initiator methionine, Lys^66^ and Lys^120^ occur in two of the four Hbb CRAC/CARC motifs. This suggests that insulin directly affects cholesterol binding to the Hb β subunit. Mb is similar to the β-chain of hemoglobin (Hbb) and has a sufficient number of ε-amino groups to be glycosylated like Hbb. Banerjee and Chakraborti (2014) have shown that glyoxal, a highly reactive oxoaldehyde, is elevated in diabetes and modifies Lys^145^ (a residue within one of two Mb CRAC/CARC motifs), suggesting that insulin may also effect Mb function.

A link between insulin and cholesterol binding is also demonstrated by the finding that, under hypoglycemic conditions, hyperinsulinism causes an increase in cholesterol binding to Hb (Tomasevic et al., 2003). Using a fluorescent lipid analog, these workers have shown that lipid internalization occurs in erythrocytes in response to elevated insulin levels. A further link between glycosylation and diabetes is the report that glycosylation *per-se* leads to blood pressure reduction in type 2 diabetics patients untreated for hypertension (Cabrales et al., 2008).

### Characterization of the Hb binding site on the erythrocyte membrane

There is a long history concerned with Hb binding sites on the red cell membrane (see Shaklai et al., 1977). Current evidence indicates that anion exchange protein “band 3,” a multipass membrane protein, is the major membrane binding site for Hb in the human erythrocyte (e.g., Chu et al., 2008). The bicarbonate/chloride exchanger “band 3” (Anion Exchanger, AE1) is an important structural protein in the erythrocyte cell membrane (van den Akker et al., 2010). Each human erythrocyte is estimated to contain 1 million copies of “band 3.” “Band 3” is also thought to play an important role in transmembrane gas exchange and function as a point of attachment for the cytoskeletons that maintain the osmotic properties of the cell (cit. van den Akker et al., 2010). The oxygenation state of Hb also plays an important role, since deoxyhemoglobin, but not oxyhemoglobin, binds “band 3” reversibly with high affinity (van den Akker et al., 2010).

**Figure 6:**
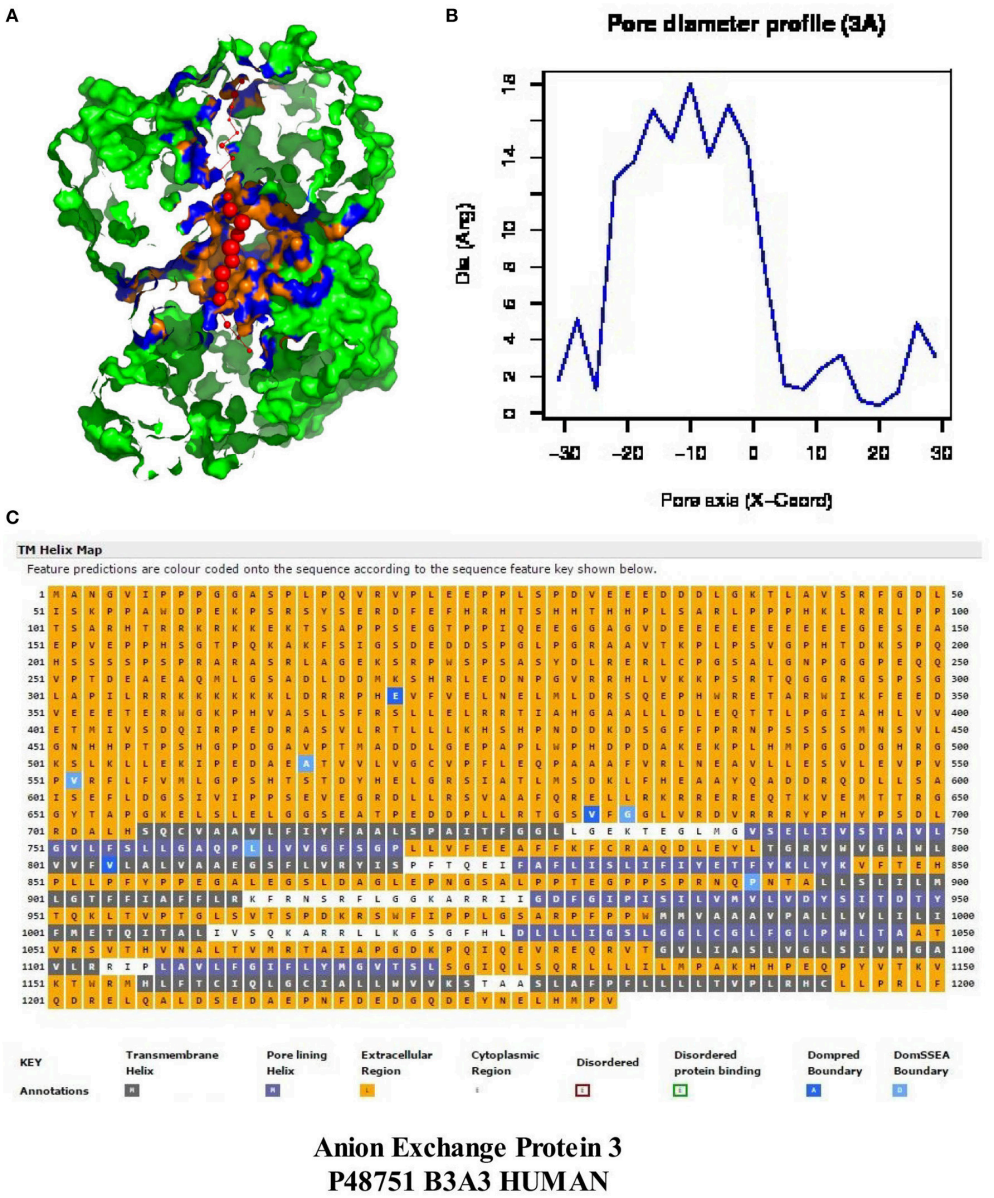
**An analysis of the MEMSAT-SVM topology and PoreWalker prediction for anion exchange “protein 3” (***Homo sapiens***, Accession #P48751)**. Panel **(A)** represents PoreWalker output for “protein 3” pdb B3A3 (top left). Panel **(B)** illustrates the pore diameter profile at 3À steps. Panel **(C)** represents the topology using the support vector machine-based TM topology predictor (see Section Methods). Transmembrane pore-lining regions are highlighted in blue, whereas transmembrane helices are highlighted in black. White sequences indicate predicted cytoplasmic regions; those highlighted in orange represent extracellular regions.

Figure 6 demonstrates that *H. sapiens* “band 3” (Accession #P48751) contains a large central cavity (Figures 6A,B) as well as 12 transmembrane helices located in the C-terminal region. The 705 N-terminal residues are extracellular with the subsequent 491 residues containing 12 closely packed helices and/or pore-lining regions. The five helices indicated by blue squares (2, 4, 6, 8, 10) are predicted to be pore-lining regions (Figure 6C). It should also be noted that about half of all transmembrane helices contain bends and other deviations often referred to as “kinks” (reviewed in Meruelo et al., 2011). As proposed by Meruelo et al. (2011), distortions in helix geometry may facilitate conformational changes required for protein function by providing sites of flexibility and can be important for positioning key residues precisely in the protein structure. Kinks that open the polar backbone to alternative hydrogen bonds often attract water molecules, thus providing a polar region within the hydrophobic core (Hall et al., 2009). TMkink analysis indicates that TM-1 of “band 3” contains a large kink (^710^A – A^723^), thus providing a potential polar-water filled region. Anion exchange protein “band 3” thus contains numerous pore-lining regions as well as a water-containing kink suggesting that the anion exchange protein (“band 3”) may merge with pore-lining regions in hemoglobin molecules to form bicarbonate/chloride exchange channels (see Hildebrand et al., 2006; Nugent and Jones, 2012 for discussion of channel/pore formation).

### Comparison of features of the cavities in Cybg, Ngb, Mb, and the Hb tetramer

**Figure 7:**
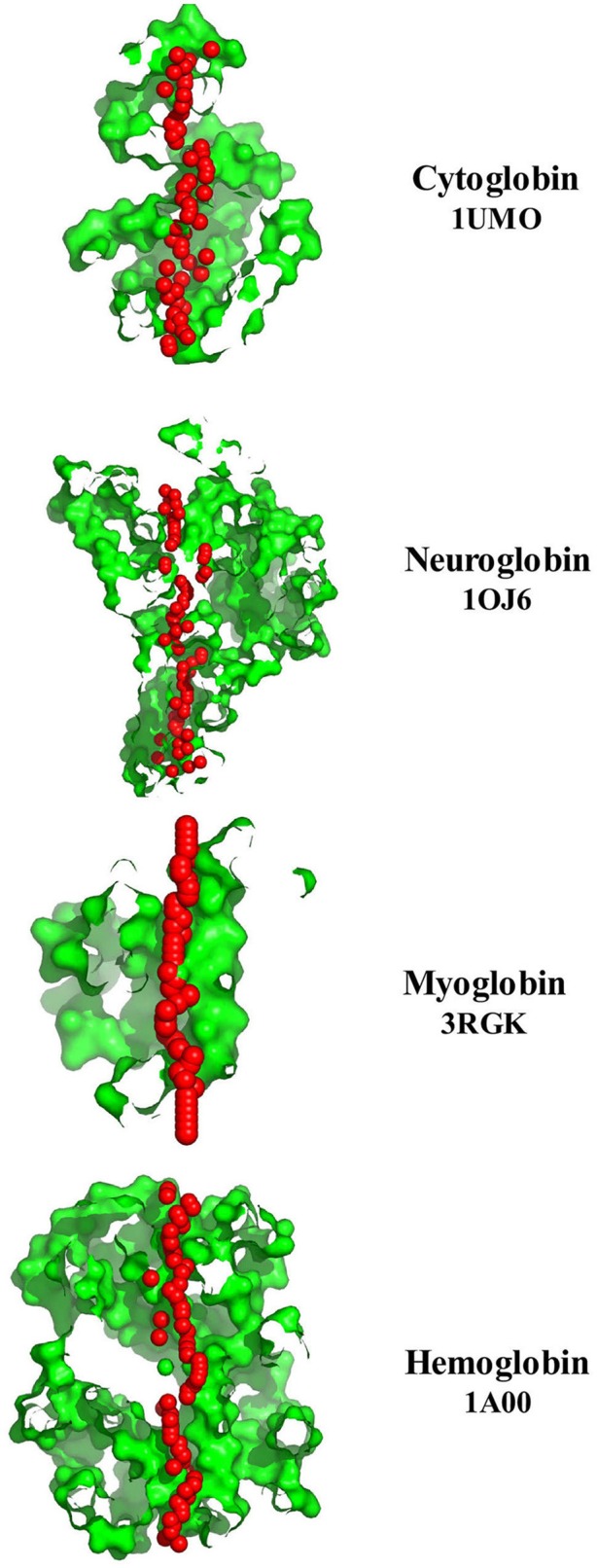
**A comparison of the pore diameter profiles for cytoglobin (upper plot, 1UMO), neuroglobin (upper middle plot, 1OJ6), myoglobin (lower middle plot, 3RGK), and hemoglobin (lower plot, 1A00) using the PoreWalker algorithm (Pellegrini-Calace et al., 2009)**. For details see Section Methods.

The regularity of the pore cavity is deduced from the positions of the pore centers calculated by PoreWalker, aimed at the optimization of the pore axis. If all the pore centers within a channel are co-linear, then the pore is linear and the cavity is symmetrical. If a pore centers in only certain areas of the channel, but not the entire channel, and are co-linear, the cavity will be non-symmetric, and the channel irregular. Figure 7 compares the protein orientation of Cybg (top), Ngb (upper middle), Mb (lower middle), and Hb (bottom) for the *XZ* plane section when Y > 0 coordinates only. Cybg exists as a dimer, Ngb and Mb are monomers and Hb is a 4-mer (ABAB). As noted above, cholesterol and other lipids have been removed prior to crystal formation. The lowest coordinate is along the pore axis (=*x*-axis) at the bottom. The red spheres represent pore centers at 1 À steps along the pore axis. Cavities traverse each globin, indicating that all four globins without bound cholesterol contain a single major continuous pore or channel.

Table 2 identifies distal and proximal Fe sites for Cybg, Ngb, Mb, and Mbb at the top of each column and underlines the cavity-lining Fe binding sites within Cybg (^113^H, heme proximal ligand), Ngb (^96^H, heme proximal ligand), Mb (^65^H, heme distal ligand), and Hbb (^64^H, heme distal ligand). The cavity-lining Fe binding site for Hba was ^88^H, heme proximal ligand (data not shown). Cavity-lining residues are defined as amino acids contributing at least one atom to the inside surface of the pore. The pore surface can be considered as a continuum made up of horizontal and vertical layers of atoms. As also shown in Table 2, the cavity-lining regions involve 57 of 190 Cybg residues, 38 of 151 of Ngb residues, 55 of the 154 Mb residues, or 35 of 144 residues in Hbb (subunit β). Residues associated with cholesterol binding (CRAC, CARC) motifs are highlighted in red. As indicated, each globin contains one or more bound cholesterol molecule within the predicted cavity and/or channel. For comparison, Table 3 compares the specific residues within the pore-lining regions that correspond to the cavity-lining residues identified by PoreWalker. Ten pore-lining residues within Cybg correspond to cavity-lining residues identified by PoreWalker. Similarly, 9 pore-lining residues are within the cavity of Ngb, and 10 and 8 residues were within the cavities of Mb and Hbb, respectively. Many of the residues identified by PoreWalker as pore-lining residues were confirmed as accessible to water (hydrophilic sites) using a water accessibility predictor (Lingwood et al., 2009) for α-helical transmembrane proteins (MPRAP).

**Table 2:** **Comparison of the predicted cavity-lining residues in cytoglobin, neuroglobin, myoglobin, and hemoglobin β using PoreWalker analysis: red highlights indicate CRAC/CARC binding sites**.

**Cytoglobin (Cybg) Fe binding sites 81, 113**		**Neuroglobin (Ngb) Fe binding sites 64, 96**		**Myoglobin (Mb) Fe binding sites 65, 94**		**Hemoglobin** β **(Mbb) Fe binding sites 64, 93**	
18	102	3	104	9	93	15	101
19			105				102
20	106	67		12	99	19	104
	109	68		13			105
23	111	71	109	14	100	22	108
24	113	72			101		109
		73	114	16	103	25	
27		74		17	104	26	112
28	122	75		20	107	27	113
30	124	78	133	21	108		115
31	125	79		24	110	30	116
34	126		136	25	111	31	117
		81	137	27		33	
37	128	82		28	114	34	131
38	129	83	139	29	115	35	132
	130	84	140		118	37	
48	131	85	141	31	119		134
49	132	86	142	32	120	52	135
	133	87			122		
52	134	88	144	43	123	55	139
53	135	89					
		90	146	64	131	61	143
54	143	91		65	132		
55	148	92		66	134	64	
		94		68	135		
67	150	95		69		68	
	151	96			138		
84	152			72	139	77	
86	153			75			
89	154			76	142		
	155				143		
92				85	146		
93	157			86			
	158			89			
96	159						
	161						
99	162						

**Table 3:** **Correlation between cavity-lining residues identified by the PoreWalker Algorithm and Pore-Lining residues predicted by MEMSAT-SVM**.

**Cytoglobin Q8WWM9**		**Neuroglobin Q9NPG2**		**Myoglobin P02144**		**Hemoglobin β P68871**	
**Cavity**	**Pore-lining**	**Cavity**	**Pore-lining**	**Cavity**	**Pore-lining**	**Cavity**	**Pore-lining**
125	125	133	133	104	105	105	
126	126		134		106		106
	127		135		107		107
128	128	136	136	107	108	108	108
129	129	137	137	108	109	109	109
130	130	139	138		110		110
131	131	140	139	110	111		111
132	132	141	140	111	112	112	112
133	133	142	141		113	113	113
134	134		142		114		114
135	135	144	143	114	115	115	115
	136		144	115	116	116	116
	137	146	145		117	117	117
	138		146	118	118		118
	139		147	119	119		119
	140		148	120	120		120

Compared to the dimeric (Cybg) or monomeric forms (Ngb and Mb), Hb is a heterotetramer consisting of two α chains and two β chains. Table 4 compares the predicted cavity-lining residues of the T state Hb heterotetromer (1GZX). Columns A and C represent the two α subunits whereas B and D correspond to the two β subunits. The CRAC/CARC motifs are highlighted in red and the pore-lining or TM helix residues are highlighted in blue. Table 4 indicates that all four tetramers contribute pore-lining and/or TM helix residues as well as cholesterol-binding (CRAC/CARC) domains to the major cavity within Hb. As shown, each heterotetramer contributes a total 29 blue-highlighted (pore-lining and TM helix) residues and 30 red-highlighted (cholesterol-binding) residues to the largest Hb cavity.

**Table 4:** **Comparisons of the predicted cavity-lining residues in the T state hemoglobin heterotetramer CRAC/CARC motifs in red highlights; pore-lining regions in blue highlights**.

**Hemoglobin α Chains 1GZX (141 residues in length)**		**Hemoglobin β Chains 1GZX (146 residues in length)**	
1	1	4	14
10	35	20	16
13	36	24	17
36	37	25	18
37	38	28	19
77	43	29	23
93	86	32	24
94	92	33	27
95	93	35	50
96	94	36	52
99	95	37	67
100	97	39	70
102	98	41	76
103	99	43	114
106	101	54	116
107	102	57	117
110	106	58	118
114	107	63	121
115	110	101	122
117	113	103	125
118	114	104	126
119	116	106	129
121	117	107	130
122	118	108	145
123	120	111	148
125	121	112	
126	122	113	
128	124	114	
129	125	115	
130	129	117	
133	132	118	
134	133	119	
137	134	120	
138	135	129	
140	139	133	
141	253	134	
	254	135	
		139	
		141	

## Discussion

Our findings indicate that cytoglobin (Cygb), neuroglobin (Ngb), myoglobin (Mb), and hemoglobin subunit β (Hbb) each contain one pore-lining channel-forming amino acid sequence in the C-terminal region as well as one or more internal cavities. In contrast, Hb subunit α (Hba) contains one transmembrane (TM) helix in the C-terminal region compared to the pore-lining structure found in the other 4 globins. The pore-lining regions, TM helices, caveolin-binding motifs and cholesterol-binding (CRAC/CARC) motifs in *H. sapiens* globins and “band 3” Anion Exchange Protein 3 are summarized in Table 5. The TM helix of Hba may serve as a membrane anchor for the Hb tetramer, accounting for the observed Ha binding to “band 3” in the erythrocyte membrane (e.g., Chu et al., 2008). Residues in the pore-lining regions of Cybd, Ngb, Mb, and Hbb also line the cavities (Table 4), indicating that the pore-lining regions of all 4 globins contribute to their respective cavities. Similarly, as evidenced by CRAC/CARC motif sequences highlighted in red in Table 2, each globin contains bound cholesterol molecules extending into the cavity. It is important to note a possibility of “false positive” results. Many proteins were shown to have multiple CRAC/CARC motifs but have not been confirmed as actual cholesterol binding domains. A variety of ion channels, including members of all major ion channel families, have been shown to be regulated both by membrane cholesterol levels and by cholesterol partitioning into cholesterol-rich membrane domains (reviewed in Levitan and Barrantes, 2012; Levitan et al., 2014). At least three mechanisms for cholesterol action have been proposed by Levitan et al. (2014): (1) specific interactions between cholesterol and channel protein, (2) cholesterol- induced changes in the physical properties of the membrane bilayer, and (3) cholesterol maintenance of scaffolds for protein-protein interactions.

**Table 5:** **Comparison of Pore-Lining regions, Caveolin-Binding Motifs and Cholesterol Binding (CRAC/CARC) Motifs in ***Homo sapiens*** neuroglobin, myoglobin, hemoglobin subunit α, and hemoglobin membrane binding sites (Anion Exchange Protein Band 3)**.

**Protein**	**Length (amino acids)**	**Pore-Lining Region**	**Caveolin Binding Motifs**	**Cholesterol Binding (CRAC/CARC) Motifs**
Cytoglobin	190	^125^K – A^140^	N.D.	^33^R – V^41^, ^119^V – K^125^
#Q8WWM9				^155^R – V^162^
Neuroglobin	151	^133^W – W^148^	^42^F – F^49^	^30^R – L^34^, ^38^L – R^37^
#Q9NPG2				^85^L – R^94^, ^152^K – V^109^
				^113^L – K^119^
Myoglobin	154	^105^L – H^120^	N.D.	^43^K – L^50^
#P02144				^140^K – l^150^
Hemoglobin Subunit α	142	^106^I – K^121^ (TM Helix)	N.D.	^41^K – L^49^
#P69905				^92^R – K^100^
Hemoglobin Subunit β	147	^105^L – K^121^	^36^Y – F^43^	^41^R – L^49^
#P68871				^66^K – L^76^
				^83^K – L^92^
				^121^K – V^127^
				^127^V – K^133^
Band 3	1232	^740^V – P^770^	^66^Y – F^73^	^316^K – L^324^, ^623^R – l^633^
Anion Exchange Protein 3		^827^F – K^885^	^774^F – F^781^	^692^Y – L^700^, ^789^Y – R^793^
#P48751		^928^G – Y^950^	^822^F – F^829^	^833^L – K^842^, ^967^K – L^975^
		^1027^D – A^1048^	^955^F – F^913^	^979^R – V^986^, ^1020^K – L^1030^
		^1107^L – L^1122^		^1154^R – L^1163^, ^1198^R – l^1205^

*N.D.: none detected*.

The cavity dimensions shown for the 4 globins in Figure 3 represent those of the lipid-free crystalline state and not those seen under physiological conditions. Cholesterol *in vivo* may serve to control cavity and/or pore/channel diameters. It is a major constituent of the plasma membrane of the eukaryotic cells essential for maintenance of membrane fluidity, thickness and dynamics, and for the compartmentalization of the lipid domains that constitute scaffolds for multiple signaling platforms (reviewed in Lingwood et al., 2009; Levitan et al., 2014). One of the most important known effects of cholesterol on lipid membranes is its cholesterol condensing effect. The surface area of a cholesterol-containing lipid bilayer is less than the sum of areas of the individual components (reviewed in Alwarawrah et al., 2010). Alwarawrah et al. (2010) have shown that the total area of phosphatidylcholine/cholesterol is primarily determined by the molecular packing in the cholesterol sterol ring region. Analysis of the area per molecule takes into account the cholesterol tilt angle and the probable incompressibility of cholesterol sterol rings. As noted by Fantini and Barrantes (2013), cholesterol is an asymmetric molecule displaying both a planar α face and a rough β face. These structural features introduce a number of possible interactions between cholesterol and membrane lipids and proteins.

Cholesterol metabolism/turnover may also play a role. For example, in the adrenal and gonad, the steroidogenic acute regulatory protein (StAR) facilitates movement of cholesterol from outer to inner mitochondrial membrane where it is converted to pregnenolone (Clark and Stocco, 1996; Miller and Strauss, 1999). Our studies (Morrill et al., 2014) have shown that subunits of the terminal enzyme in the human mitochondrial electron transfer chain (cytochrome c oxidase) contain both cholesterol (CRAC/CARC) binding motifs and leucine-rich repeats (LRRs), the latter common to plant steroid receptors (Dolan et al., 2007). Since plant steroids such as ouabain have also been identified in humans (Blaustein, 2014), the presence of steroid regulatory sites (LRRs) within the cytochrome c oxidase complex (reviewed in Morrill et al., 2014) may reflect a unique StAR protein-LRR response system in vertebrates. This is consistent with the subsequent experiments by Rosenhouse-Dantsker et al. (2013) that indicate cholesterol binding and transfer activities are distinct from the ability to induce steroidogenesis. Rosenhouse-Dantsker et al. (2013) have further identified two putative cholesterol-binding regions in inwardly rectifying (Kir) channels that suggest the existence of a novel cholesterol binding motif (Rosenhouse-Dantsker et al., 2013). Changes in erythrocyte membrane cholesterol have been shown to parallel changes in membrane surface area, as calculated from changes in osmotic fragility, with a 0.22% variation in surface area per 1.0% variation in cholesterol content (Cooper et al., 1975).

Cholesterol-dependent variations in surface area would, in turn, affect both channel formation and function. The introduction of cholesterol via both CRAC/CARC (cholesterol binding) motifs and caveolin binding domains (Figure 5) could align the cavities and facilitate cross-membrane penetration of small uncharged molecules.

Cholesterol is also required to maintain the signaling capacity of lipid rafts (see Simons and Toomre, 2000; Fielding and Fielding, 2004; Grouleff et al., 2015). Sengupta has shown (2012) that cholesterol modulates the depth as well as the orientation of caveolin-1 binding to membranes and that cholesterol stabilizes the more open conformations of caveolin-1. Sengupta speculates (2012) that the binding modes and open conformations could be responsible for inducing morphological changes in the bilayer. Both cavity formation and ligand movement within the various globins may thus be regulated by cholesterol. Pulsed laser studies by Tomita et al. (2009) have identified 3 cavities that play specific roles in controlling ligand migration and binding in myoglobin. Tomita et al. (2009) predict that the migration of the CO molecule into any cavity induces structural changes of the amino acid residues around each cavity, which results in the expansion of the cavity with a “breathing” motion. This sequential motion of the ligand and the cavity suggests a “self-opening mechanism of the ligand migration channel by induced fit” (Tomita et al. (2009). In addition, studies by Savino et al. (2009) have mapped a constellation of sites hosting up to 6 xenon atoms sites in human recombinant using Hba and Mb. The pattern of internal cavities accessibility and affinity for xenon suggest a different role for ligand migration in Hb compared to Mb.

## Conclusions

Computational analysis of protein structure indicates that cytoglobin (Cygb), neuroglobin (Ngb), myoglobin (Mb), and hemoglobin subunit β (Hbb) each contain one pore-lining channel-forming amino acid sequence in the C-terminal region as well as one or more internal cavities. The findings described both here and elsewhere suggest that changes in ligand migration may in large part reflect changes in cholesterol-rich microdomains that occur in living cells. Cholesterol appears to be involved in a variety of physiological functions in addition to regulating ion channels and membrane dynamics. Cholesterol may serve both as a steroid hormone precursor in mitochondria (Clark and Stocco, 1996; Miller and Strauss, 1999) as well as structural elements of ion channels and as control elements (Simons and Toomre, 2000; Fielding and Fielding, 2004; Grouleff et al., 2015) in multiple response systems.

## Author contributions

GM and AK conceived of the study and analyzed the data. GM wrote the manuscript with input from AK. The authors thank Dr. R. K. Gupta for valuable discussions. All authors read and approved the final manuscript.

### Conflict of interest statement

The authors declare that the research was conducted in the absence of any commercial or financial relationships that could be construed as a potential conflict of interest.
